# A Case of Trazodone Overdose Successfully Rescued With Lipid Emulsion Therapy

**DOI:** 10.7759/cureus.10864

**Published:** 2020-10-09

**Authors:** Roger Taylor, Joshua Burg, Jeff Mullen

**Affiliations:** 1 Emergency Medicine, Charleston Area Medical Center, Charleston, USA

**Keywords:** trazadone, lipid emulsion therapy, overdose, toxicology

## Abstract

Trazodone is a very common medication prescribed to patients who suffer from insomnia. The toxic effects of trazodone remain ill-defined with no current known antidote therapy. Lipid emulsion therapy has been described as general rescue therapy in toxicology. Unfortunately, only select substance overdoses respond to lipid emulsion therapy. The authors present a unique application of lipid emulsion therapy in a post-cardiac arrest situation involving a trazodone overdose.

## Introduction

Overdoses of medications and various substances occur daily in emergency departments worldwide. The ability to identify toxicity and emergent side effects is crucial in order to initiate an appropriate response therapy. Several toxic overdoses encountered in toxicology and emergency medicine do not have immediate reversal antidotes. One such medication is trazodone. The authors present a unique application of lipid emulsion therapy used in a trazodone overdose.

## Case presentation

A 54-year-old male presents to the emergency department via emergency medical services (EMS) after a suicide attempt. The patient had reported taking twenty trazodone 100 mg tablets three hours before arrival. He denied any other coingestants, including alcohol and other illicit drugs. He didn't complain about any symptoms other than feeling slightly fatigued. The patient's past medical history was significant for anxiety, depression, coronary artery disease (CAD), congestive heart failure (CHF) with a known left ventricular ejection fraction (LVEF) of 45-50%, chronic obstructive pulmonary disease (COPD), diabetes type II, and tobacco use. He had a prior cardiac catheterization with mild CAD the year prior, and he was treated with medical management. Surgical history included a prior lumbar discectomy. No pertinent family history was reported. Social history was remarkable for tobacco use, as previously noted as well as social alcohol use. The patient denied any recreational drug use. His vital signs were within normal limits on arrival: temperature 37.2C, respiratory rate 16, heart rate 92 bpm, blood pressure 126/84 mmHg, and oxygen saturation 100%. The physical exam was generally unremarkable. He spoke in clear sentences with no observable neurological deficits on the initial presentation. Suicide precautions, including one-to-one bedside attendant, were initiated in the emergency department. No specific resuscitative interventions were immediately pursued by the emergency department staff.

**Figure 1:**
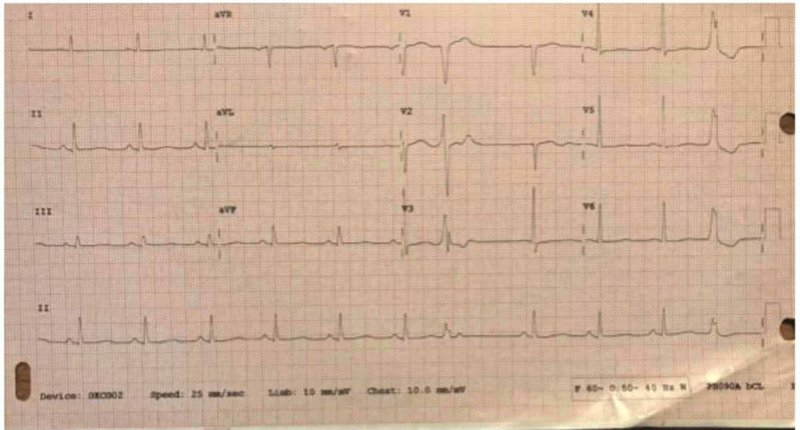
EKG performed on arrival at 15:48 EKG shows sinus rhythm at 67 beats per minute. Two premature ventricular complexes are noted. Normal PR and QRS intervals of 168 msec and 94 msec, respectively. QTc interval prolonged at 499 msec.

Laboratory workup was obtained and revealed a white blood cell count of 3.7, x 10^9^/L, hemoglobin 13.8 g/dL, hematocrit 39.7%, and platelets 100 x 10^9^/L. Chemistry panel was remarkable for sodium 135 mmol/L, potassium 3.0 mmol/L, chloride 97 mmol/L, bicarbonate 33 mmol/L, blood urea nitrogen 11 mg/dL, creatinine 0.8 mg/dL, and glucose 357 mg/dL. Subsequently, oral potassium chloride 40 mEq was given. The anion gap was five. Venous blood gas (VBG) performed with pH 7.36, pO2 23 mmHg, pCO2 56 mmHg, bicarbonate 31 mEq/L. VBG was interpreted as chronically stable hypercapnia consistent with the patient's known tobacco use and COPD. The thyroid-stimulating hormone level was 1.66 mU/L. Serum toxicology, including salicylate, acetaminophen, qualitative tricyclic antidepressants (TCA), and ethanol levels, all noted to be undetectable. A urine drug screen was negative for opiates, benzodiazepines, marijuana, ecstasy, amphetamines, barbiturates, cocaine, tetrahydrocannabinol, methadone, and phencyclidine. A urinalysis was also unremarkable. Initial EKG showed sinus rhythm at 67 beats per minute with an isolated premature ventricular contraction, narrow QRS with QRS duration noted to be 94 milliseconds. QTc interval was prolonged at 499 milliseconds (Figure 1).

The patient was observed in the emergency department for approximately two hours with plans for medical admission. However, the patient was noted to have an acute change in clinical status by the one-to-one attendant. The patient suddenly was found to be unresponsive with no palpable pulse. Cardiopulmonary resuscitation (CPR) was immediately started, and advanced cardiac life support (ACLS) protocol followed. The patient was intubated during this time. Telemetry strips revealed that the patient was in polymorphic ventricular tachycardia (torsades de pointes) just prior to the unresponsive episode. Magnesium sulfate 4 grams intravenously (IV) was immediately administered. The patient received a total of two defibrillation attempts at 200J, epinephrine 1 mg IV, and amiodarone 150 mg IV within a four-minute period. After considering possible sodium channel blockade effect, prolonged QTc, and newly widened QRS complexes, a total of sodium bicarbonate 350 mEq was also given as IV pushes. Return of spontaneous circulation (ROSC) was achieved, and the patient was started on** **dextrose 5% in water (D5W) plus sodium bicarbonate 150 mEq at 200 mL/hr as well as an amiodarone infusion at 1 mg/min.

**Figure 2:**
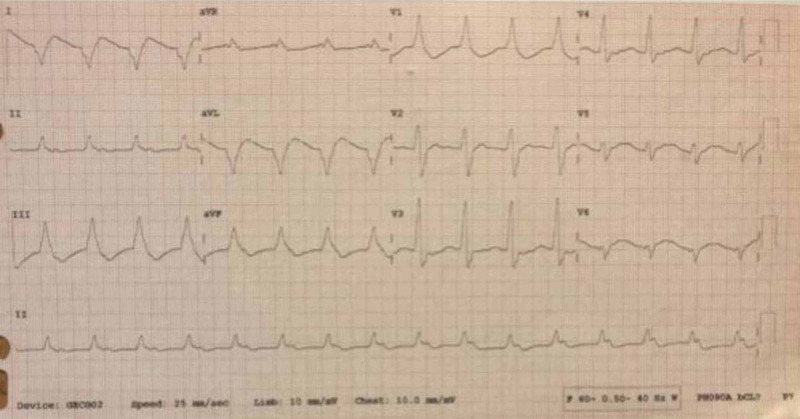
EKG performed at 18:01 after cardiac arrest and before administration of 20% lipid emulsion therapy QRS is widened at 192 msec and QTc severely is prolonged at 539 msec.

**Figure 3:**
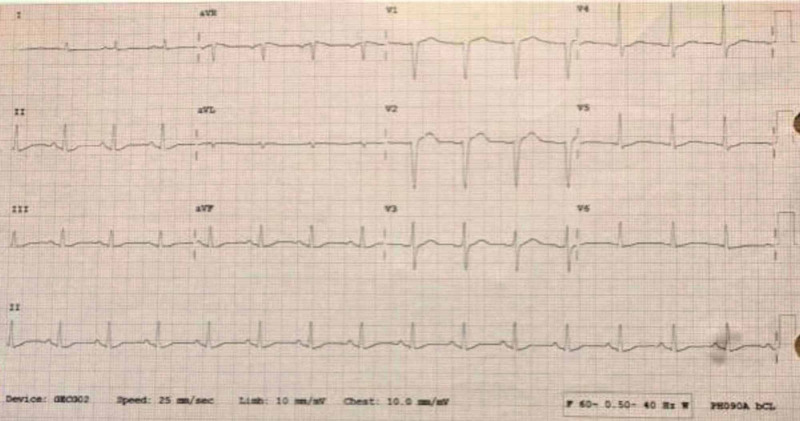
EKG performed at 18:03 after administration of 20% lipid emulsion bolus EKG shows a sinus rhythm at 90 beats per minute. PR 156 msec. QRS has improved to 102 msec and normalized. Additionally, QTc is improved to 485 msec.

An immediate post-ROSC EKG revealed a prolonged QTC of 539 with widened QRS of 192 (Figure 2). Twenty percent (20%) lipid emulsion was administered as a bolus of 1 mL/kg, and an infusion of 0.25 mL/kg/hr initiated. A repeat EKG revealed marked improvement in EKG findings with QTC improving to 485 and QRS narrowing to 102 within two minutes (Figure 3).

The patient was subsequently admitted to the intensive care unit (ICU). A trazodone level returned a few days later and was markedly elevated at 4838 mg/dl. The patient did receive a cardiology consultation while in the ICU. A transthoracic echo was performed with no new findings evident. The patient's LVEF was noted to be 45-50%, consistent with the patient’s known history of CHF. The patient was extubated the following morning after admission to the ICU with no neurological deficits noted. The patient continued to have suicidal ideations. The patient was transitioned to the care of psychiatric services after cardiology planned no interventions. The patient was deemed psychiatrically stable for discharge after a 10-day admission.

## Discussion

Trazodone has a variety of mechanisms and is structurally different from other antidepressants. The primary mechanism of action (MOA) is via serotonin reuptake inhibition, as well as through antagonism of postsynaptic serotonin type two receptors [1]. Peak plasma levels of trazodone occur around one to two hours after ingestion. It is protein-bound and is oxidized by cytochrome P450 in the liver [2]. Trazodone has other mechanisms and is notably a potent alpha-receptor blocker with greater affinity for the alpha-1 receptor [1]. This causes increased concern for orthostatic hypotension and typically the desired sedation side effect for those patients suffering from insomnia. It is also one of the most common drugs to cause priapism at around 1-10 per 10,000 patients [2]. Trazodone is rarely arrhythmogenic at therapeutic doses but can cause arrhythmias in patients with known conduction or ischemic disease [3-7].

Management of trazodone overdose generally is supportive. Cardiac monitoring is required, and serial EKGs can assist in monitoring conduction intervals. If patients present within a one-hour window, activated charcoal can be considered [2]. Hypotension can be treated with crystalloid boluses and, if necessary, vasopressors. Seizures can be treated with benzodiazepines. If QTc prolongation or torsades de pointes occurs, this can be treated with defibrillation and magnesium sulfate administration [3]. Lipid emulsion therapy is routinely not recommended in trazodone overdoses.

Lipid emulsion content varies but generally contains soybean oil or egg yolk lipids [2]. Its original use was for local anesthetic toxicity [1]. The MOA is to absorb the active fat-soluble drug content but also to form a fat-soluble substrate that improves myocyte function and intracellular calcium levels [5]. Lipid emulsion therapy has increasingly gained favor in overdoses of beta-blockers, calcium channel blockers, and antidepressant medications in toxicology literature [2, 5]. One case is discussed in anesthesia literature, which described the utility of lipid emulsion therapy in terminating prolonged ventricular tachycardia/fibrillation secondary to bupropion and lamotrigine overdose [5]. There are very few cases describing the use of lipid emulsion therapy in trazodone overdose [8-9]. However, to date, the authors have not found case reports of lipid emulsion therapy in post-cardiac arrest cases related to trazodone overdose. There were no reports specifically related to the rapid improvement of the QTc interval post-lipid emulsion in trazodone overdoses.

Dosing involves giving a 20% lipid emulsion as a bolus of 1-1.5 ml/kg over 2-3 minutes and then an infusion at 0.25 ml/kg/hr. Repeat bolus doses can be given as well as increasing the infusion rate up to 10 ml/kg maximum over 30 minutes [2]. Other various dosing regimens have been described [8-9]. This case does add to the growing literature on the successful utility of lipid emulsion therapy in trazodone overdoses.

## Conclusions

Overall, this case exemplifies the utility of lipid emulsion therapy in the management of trazodone overdose. The use of lipid emulsion therapy seemed to have a rapid effect on QTc prolongation which terminated the arrhythmogenic effect of trazodone toxicity. This perhaps prevented further clinical destabilization in the patient's clinical course. This case provides evidence that lipid emulsion therapy may offer a potential reversal of cardiac conduction abnormality associated with trazodone toxicity. Further research will be required to elucidate the role of lipid emulsion therapy in trazodone toxicity.
